# Hyperspectral Infrared Observations of Arctic Snow, Sea Ice, and Non-Frozen Ocean from the RV Polarstern during the MOSAiC Expedition October 2019 to September 2020

**DOI:** 10.3390/s23125755

**Published:** 2023-06-20

**Authors:** Ester Nikolla, Robert Knuteson, Jonathan Gero

**Affiliations:** Space Science and Engineering Center, University of Wisconsin-Madison, Madison, WI 53706, USA; nikolla@wisc.edu (E.N.); jonathan.gero@ssec.wisc.edu (J.G.)

**Keywords:** Arctic, CrIS, M-AERI, MOSAiC, NUCAPS, RV Polarstern, validation

## Abstract

This study highlights hyperspectral infrared observations from the Marine-Atmospheric Emitted Radiance Interferometer (M-AERI) collected as part of the Department of Energy (DOE) Atmospheric Radiation Measurement (ARM) Mobile Facility (AMF) deployment on the icebreaker RV Polarstern during the Multidisciplinary drifting Observatory for the Study of Arctic Climate (MOSAiC) expedition from October 2019 to September 2020. The ARM M-AERI directly measures the infrared radiance emission spectrum between 520 cm^−1^ and 3000 cm^−1^ (19.2–3.3 μm) at 0.5 cm^−1^ spectral resolution. These ship-based observations provide a valuable set of radiance data for the modeling of snow/ice infrared emission as well as validation data for the assessment of satellite soundings. Remote sensing using hyperspectral infrared observations provides valuable information on sea surface properties (skin temperature and infrared emissivity), near-surface air temperature, and temperature lapse rate in the lowest kilometer. Comparison of the M-AERI observations with those from the DOE ARM meteorological tower and downlooking infrared thermometer are generally in good agreement with some notable differences. Operational satellite soundings from the NOAA-20 satellite were also assessed using ARM radiosondes launched from the RV Polarstern and measurements of the infrared snow surface emission from the M-AERI showing reasonable agreement.

## 1. Introduction

Arctic research over the past two decades has shown warming surface temperatures, increases in humidity and thinning sea ice [1,2]. The scientific community has identified the need to observe, understand, and model the changes [3]. A major international effort was conducted to make accurate scientific observations during an annual cycle of atmospheric properties, processes, and interactions while drifting with the sea ice across the central Arctic during the Multidisciplinary drifting Observatory for the Study of Arctic Climate (MOSAiC) expedition from October 2019 to September 2020 [4,5,6,7,8]. The MOSAiC expedition was a collaborative effort among 20 nations that collected many unique observations. The collaborative data are available on several archives including the MOSAiC data archive at the German PANGAEA Data Publisher, the UK Polar Data Centre, the British Oceanographic Data Center, the US Arctic Data Center, and the US Department of Energy (DOE) Atmospheric Radiation Measurement (ARM) data archive [5].

The MOSAiC expedition has been put into a climatological context using satellite remote sensing observations and model reanalysis over the past decade for air temperature, sea ice concentration and thickness, snow depth, precipitation, and other meteorological processes [9]. The MOSAiC campaign experienced a wide variety of conditions during the annual cycle. A suite of surface-based, active and passive remote sensing systems was deployed to monitor the seasonal evolution of snow and ice properties including temperature and emissivity [10]. One of the major contributions to the MOSAiC expedition was provided by the U.S. DOE ARM through the deployment of a mobile facility (AMF2) onboard the RV Polarstern and on the surrounding sea ice [5,11,12]. Built in 2008, the second DOE ARM mobile facility, AMF2, is a flexible suite of in situ and remote sensing instruments that have supported numerous field campaigns from Antarctica to the Arctic in a combination of ship-based and land-based environments [13]. During the MOSAiC campaign, the sensors from the AMF2 were distributed between the RV Polarstern and the meteorological observing sites on the ice pack a short distance from the ship. The RV Polarstern P-deck, above and directly aft of the bridge, offered a suitable location for a large collection of sky-observing systems, some of which viewed vertically, while others viewed the sky over a range of angles [5].

**Figure 1:**
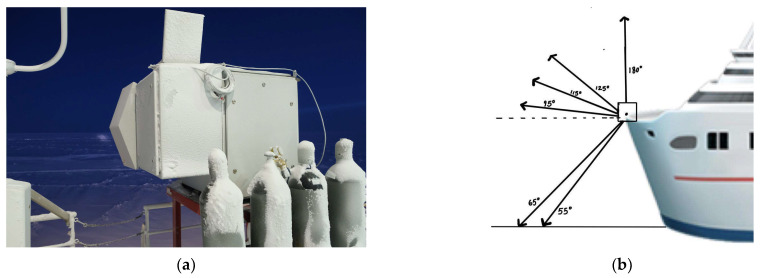
(**a**) Photo of DOE ARM M-AERI installed on the RV Polarstern P-deck [Image courtesy of the U.S. Department of Energy Atmospheric Radiation Measurement (ARM) user facility.] and (**b**) illustration showing M-AERI viewing angles of sea surface and sky.

The Marine Atmospheric Emitted Radiance Interferometer (M-AERI) operated continuously from the RV Polarstern P-deck measuring spectral infrared emitted radiance from the sky and sea surface, including infrared emission from trace gases and water/ice clouds [5,14]. A co-author of this manuscript (J. Gero) is the ARM instrument mentor for the M-AERI sensor. Dr. Gero was responsible for the M-AERI installation on the RV Polarstern with a scan view at the six view angles shown in Figure 1b. The ARM M-AERI directly measures the infrared radiance emission spectrum between 520 cm^−1^ and 3000 cm^−1^ (19.2–3.3 μm) with spectral sampling of ~0.5 cm^−1^ in a narrow cone along a slant path controlled by a rotating scene mirror [15]. The zenith viewing AERI sensor was developed at the University of Wisconsin-Madison Space Science and Engineering Center in the 1990s and licensed to ABB, Inc. of Quebec, Canada for commercial manufacture [16,17]. Several successful algorithms have been developed for the retrieval of planetary boundary layer temperature and water vapor with relatively high vertical and temporal resolution [18,19,20]. An early prototype of the M-AERI was used to measure the ocean infrared emissivity spectrum in the Gulf of Mexico from the LUMCON ship Pelican in 1996 [21]. A zenith looking AERI instrument was deployed previously on an icebreaker in the Arctic during the SHEBA campaign in 1998 for the measurement of the water vapor continuum in the far infrared [22]. The MOSAiC field campaign represents the return of the AERI to the Arctic sea ice in a Marine-AERI configuration with observations of the infrared spectral emission from the sea surface, clouds and sky. This scanning configuration led to unique observations from the M-AERI which capture the fine scale temperature stratification from the sea surface into the lowest kilometer above the RV Polarstern.

This paper describes some of the remote sensing data available from the ARM AMF2 M-AERI on-board the RV Polarstern during the period from October 2019 to September 2020 while frozen into the Arctic sea ice. The M-AERI infrared observations have been used to determine the change in air temperature with height in the lowest levels of the atmosphere. The M-AERI conducted routine angular scanning of the atmosphere at zenith (180° from nadir), nearly horizontal (95° from nadir), and two intermediate angles (125° and 115° from nadir). The accurate radiometric calibration of the M-AERI infrared emission spectrum, using high emissivity blackbodies, allows for the determination of air temperature at a range starting about 30 m from the instrument and reaching a height of several kilometers [23]. Since the M-AERI was mounted on the upper P-deck of the RV Polarstern the measurements provide complimentary information to the observations on the ice pack located away from the ship. In particular, the temperature profile inversion in the kilometer above the RV Polarstern was monitored using the M-AERI with higher temporal resolution (less than 10 min intervals) than was possible from radiosondes at 6 h intervals. For a cold, dry, clear sky period, the infrared surface emissivity has been determined from the M-AERI spectral radiance observations. These ship-based observations provide a valuable set of truth data for the modeling of snow/ice infrared emissivity [24] as well as validation data for the assessment of satellite soundings [25]. In particular, the NOAA-unique combined atmospheric processing system (NUCAPS) provides routine temperature and water vapor soundings over the Arctic region. However, the presence of low clouds over the polar sea ice and surrounding Arctic ocean make the validation of the satellite sounding profiles in the lowest layers of the atmosphere challenging. The MOSAiC campaign provides some of the highest quality validation datasets in a region that is typically deficient in ground-truth observations. The mid- and far-infrared radiance observations of the. M-AERI are also relevant to inform the NASA Polar Radiant Energy in the Far Infrared Experiment (PREFIRE) [26] and the ESA Far-infrared Outgoing Radiation Understanding and Monitoring (FORUM) [27] missions, which will be launched in the coming years to better understand the Earth’s energy budget in the polar regions.

## 2. Materials and Methods

The M-AERI sensor [ABB Inc, Saint-Laurent, QC, Canada] uses a Fourier transform spectrometer (FTS) to measure the emitted radiance in to broad spectral bands in the thermal infrared at high spectral resolution [15]. The sensor is divided into two sections: (1) an external scene scanning compartment; and (2) a temperature/humidity controlled enclosure for the FTS and detector aft optics [16]. The two sections are separated by a ZnSe window. The external compartment is composed of a gold-coated 45° scene mirror, two high emissivity blackbody cavities, and a vertical slot for sky and ocean/ice viewing. One of the blackbodies is controlled to a warm temperature (typically 60 °C), while the “ambient” blackbody is not controlled and typically follows the external air temperature. The scene viewing mirror steps through a series of sky and Earth view angles followed by views of the two blackbody cavities in a continuous cycle that repeats about every 2 min. This continuous calibration allows the M-AERI to maintain a high calibration accuracy over long time periods and in remote locations [17].

**Figure 2:**
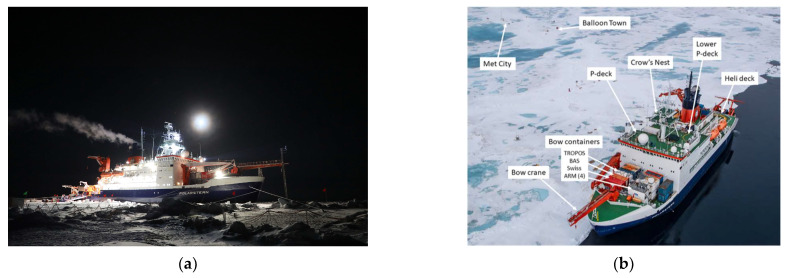
(**a**) RV Polarstern frozen into arctic sea ice during the MOSAiC campaign. [Image courtesy of the U.S. Department of Energy Atmospheric Radiation Measurement (ARM) user facility.] (**b**) MOSAiC sensor configuration. The M-AERI is on the port-side upper P-deck in this photo. [Photo credit: Lianna Nixon. DOI: https://doi.org/10.1525/elementa.2021.00060 f3 (accessed on 30 April 2023)].

**Figure 3:**
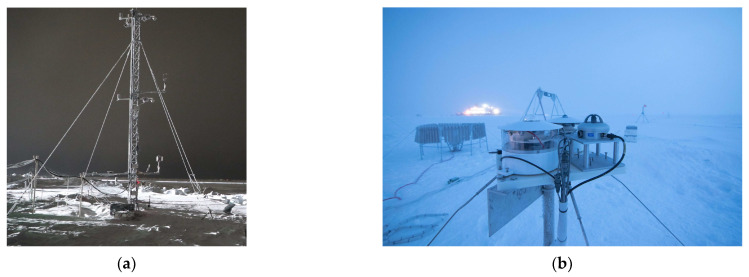
(**a**) ARM Meteorological tower and (**b**) ARM surface radiation station. [Images courtesy of the U.S. Department of Energy Atmospheric Radiation Measurement (ARM) user facility].

**Figure 4:**
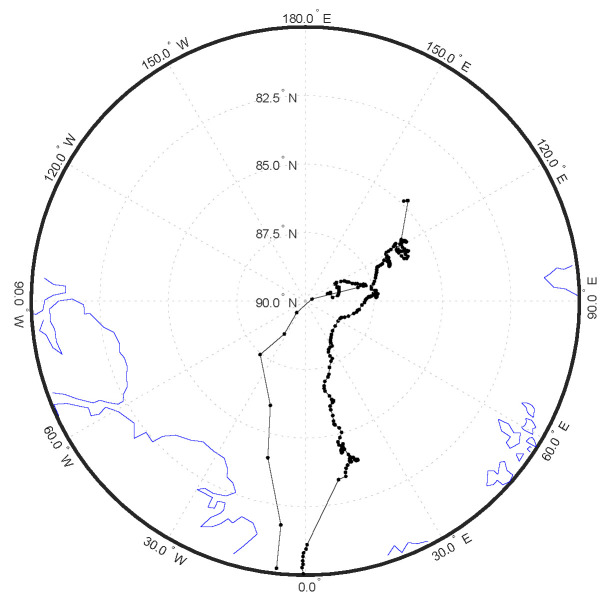
Daily location of the RV Polarstern and M-AERI during MOSAiC.

**Figure 5:**
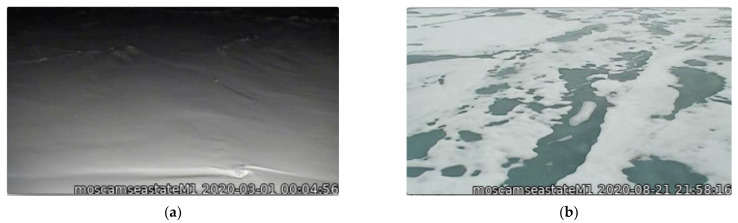
M-AERI video camera (**a**) 1 March 2020 snow surface and (**b**) 21 August 2020 water/ice slush.

The MOSAiC campaign data used in this study were obtained from the DOE ARM archive (www.arm.gov/data (accessed on 1 June 2022)). This includes: M-AERI brightness temperatures, meteorological observations of meteorological air temperature and humidity, wind speed and direction, air pressure, surface skin temperature, and radiosonde profiles [28]. Figure 2 shows photographs of the RV Polarstern in the Arctic sea ice during the MOSAiC campaign. Figure 3 shows photographs of the ARM Met tower and ARM surface radiation station on the sea ice. Figure 1 illustrates the M-AERI instrument configuration at the railing of the RV Polarstern port-side upper P-deck. An AXIS Communications Q6055-S video camera [Axis Communications Inc., Chelmsford, MA 01824, United States], with the ARM data product name “moscamseastate”, captured the image of the sea surface also viewed by the M-AERI. The frame of the video camera image is larger than the projected footprint of the M-AERI on the sea surface; however, the video camera was aligned so that the M-AERI downlooking footprint is approximately in the center of the image frame. Figure 4 shows the locations of the RV Polarstern where M-AERI observations were collected during the MOSAiC campaign. A detailed description of the RV Polarstern ship track location and timing can be found in Krumpen et al. [9]. A qualitative description of environmental conditions derived from the moscam video is provided in Table 1. Two case studies were selected from the MOSAiC campaign for detailed analysis. The first case is from early March 2020 during the coldest and most cloud-free time period of the campaign (RV Polarstern location 88.2° N, 31.4° E). The second case is in August 2020 (87.75° N, 104.3° E) and is representative of melting conditions. Figure 5 shows images from the moscam video camera showing the M-AERI slant view to the sea surface for each case. The March case indicates darkness with snow covering the sea ice, whereas the August case indicates overcast daylight with a combination of slushy snow and small melt ponds.

**Figure A1:**
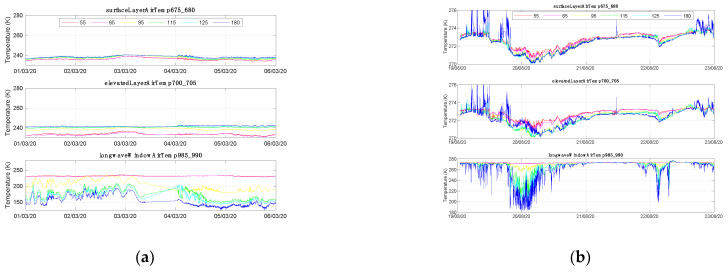
(**a**) M-AERI longwave brightness temperature time series for 1–5 March 2020, and (**b**) 19–22 August 2020.

This study makes extensive use of the ARM M-AERI summary netCDF files for each UTC date [29]. In particular, the variables surfaceLayerAirTemp675_680, elevatedLayerAirTemp700_705, and longwaveWindowAirTemp985_990 represent the mean brightness temperature of the observed infrared radiance spectrum over the wavenumbers indicated (675–680 cm^−1^ in the opaque 15 μm CO_2_ band, 700–705 cm^−1^ in the wing of the 15 μm CO_2_ band, and 985–990 cm^−1^ in the transparent atmospheric infrared window region). Note that a spectral average over a five wavenumber range reduces the noise on the mean by about a factor of three over noise on a single spectral channel. With that noise reduction, no additional temporal smoothing was necessary for the analysis of the timeseries data. Figure A1 in Appendix A illustrates the time series of the M-AERI brightness temperature for the six M-AERI view angles for each case study period. The surfaceLayerAirTemp675_680 is an estimate of the mean air temperature at a range between 30 and 100 m from the sensor. The difference between the M-AERI elevatedLayerAirTemp700_705 and the surfaceLayerAirTemp675_680 brightness temperatures can be used to estimate the temperature lapse rate in the boundary layer. The surfaceLayerAirTemp675_680 of the four M-AERI scene view angles above the horizon can be interpreted as the air temperature along those slant views within 100 m of the RV Polarstern (95°, 115°, 125°, and 180° from nadir). The longwaveWindowAirTemp985_990 is an estimate of the brightness temperature of the far-field scene view for each angle. Since the M-AERI is close to the sea surface, the longwaveWindowAirTemp985_990 values for downlooking angles (55° and 65° from nadir) provide a direct measure of the emission from the sea surface plus the diffuse reflection of downwelling infrared radiation at that wavelength. For the uplooking angles (95, 115, 125, and 180 from nadir), the longwaveWindowAirTemp985_990 provides a measure of the sky brightness temperature along the slant angle.

The derivation of infrared spectral emissivity presented in this paper uses the longwave band of the M-AERI sensor contained in the product file “mosaerich1” [14]. The method used to separate skin temperature and infrared emissivity using the M-AERI observations is based on the formulation described in [30]. Since the snow surface is assumed to be diffuse, the downwelling flux in this method is represented by the M-AERI sky view at 125° from nadir for the emissivity determination of both the 55° view and the 65° view. For the ship-based M-AERI, we assume that the radiance emission of the surface emission within the small M-AERI footprint (~30 cm) is homogeneous so that the weight is set to unity. For a window channel, we can also assume that the transmission is unity. In this formulation, the skin temperature is varied to obtain the emissivity spectrum that has the fewest spectral contributions from atmospheric absorption lines. This physical constraint allows us to determine a skin temperature from the M-AERI infrared spectra whenever the sky is clear. Using this optimal skin temperature, we then derive the infrared surface emissivity spectrum that is consistent with the formulation of [30]. This is the method used in Section 3.2.2 of this paper.

Under the conditions where the sensor is close to the surface boundary and a transparent window radiance is used, the observed radiance of the surface view contains the sum of the gray body emission at the surface skin temperature plus the reflection of the downwelling atmospheric flux into the sensor field of view. This is illustrated in Equation (1), where e is the infrared surface directional emissivity, R^up^ is the upwelling radiance from the surface, R^dwn^ is the downwelling flux from the sky, and B is the Planck function.

R^up^ = e B(T_s_) + (1 − e) R^dwn^ (1)

Assuming we know the surface emissivity at the wavelength of interest, we can derive the skin temperature at any time from M-AERI radiance observations using Equation (2).

T_s_ = B^−1^ ((R^up^ − (1 − e) R^dwn^)/e) (2)

where B^−1^ is the inverse Planck function. The window channel radiance average between 985 and 990 cm^−1^ (10.15–10.10 μm) was used for the determination of the skin temperature because the emissivity is relatively high and the atmospheric transmission is close to unity for the short path from the M-AERI to the sea surface. For this study, a value of infrared emissivity of 0.998 was chosen based on the analysis shown in Section 3.2.2 of this paper for the March 2020 case study. This is very close to the value of 0.996 used for the conversion of radiance data to skin temperature from a helicopter borne radiometer survey of the MOSAiC site [31]. The high value of the infrared emissivity at this window channel wavenumber range means that the infrared skin temperature is close to the observed brightness temperature and the correction for the downwelling atmosphere will be small but is still taken into account. This is the method used in Section 3.2.1 of this paper.

The ARM MET tower data were obtained from the ARM archive as the product name “mosoasmet” [32]. The variable “temperature_ambient” contains the standard 2 m height near-surface air temperature. The MET tower is also the source for wind speed and direction information near the sea surface. The ARM downlooking infrared radiometer (IRT) was obtained from the ARM archive as the product name “mosgndirt” [33]. The variable “sfc_ir_temp” contains the surface skin temperature derived from the IRT radiance observation. The ARM radiosonde data were obtained from the ARM archive as the product name “sondewnpn” [34]. The ARM ceilometer data were obtained from the ARM archive as the product names “mosceil”, “mosceil10m”, and “mosceilpblht” [35].

For the satellite operational sounding retrievals, the NOAA Unique Combined Atmospheric Processing System (NUCAPS) was used to provide retrieved profiles of temperature and water vapor. The NUCAPS Environmental Data Records (EDR) products were obtained from the Comprehensive Large Array-Data Stewardship System (CLASS) for archive non-real time users. The NUCAPS version 2.0 product files used in this study were acquired from www.class.noaa.gov (accessed on 1 June 2022). The product file name used for the surface skin temperature and emissivity comparison to M-AERI on 01 March 2020 was “NUCAPS-EDR_v2r0_j01_s202003010055430_e202003010056130_c202003010120210.nc”. The variables used from this product file were Skin_Temperature, FG_IR_Surface_Emis, and IR_Surface_Emis.

## 3. Results

The following sections describe near-surface air temperature measurements, surface skin temperature and infrared emissivity measurements, and satellite sounder profile validation.

### 3.1. Near-Surface Air Temperature

**Figure 6:**
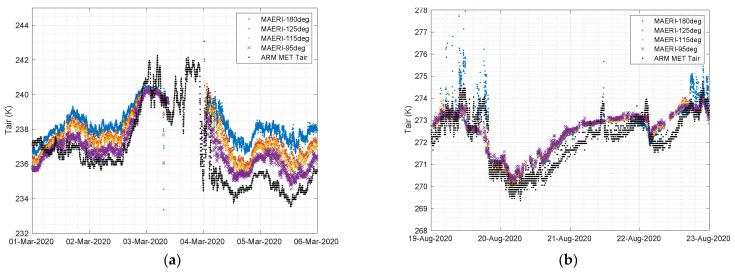
M-AERI air temperature at four uplooking slant angles compared to ARM MET air temperature for (**a**) 1–5 March 2020 and (**b**) 19–22 August 2020.

The M-AERI summary product variable “surfaceLayerAirTemp675_680” contains brightness temperatures for all view angles at 14.7 μm. Figure 6 compares the M-AERI air temperature estimates at the four uplooking slant angles to ARM MET tower air temperature for the March and August 2020 cases, which were the coldest and warmest time periods of the MOSAiC campaign. Note that the M-AERI view angle of 95° above nadir is close to the height of the P-deck on the RV Polarstern, while the 180° view is zenith above the ship. The mean path length along the line of sight is between 30 and 100 m. The other two angles are intermediate slant views at 115 and 125° from nadir. Refer to the discussion section for more details.

### 3.2. Surface Skin Temperature and Infrared Emissivity

Using Equations (1) and (2), the surface skin temperature and infrared emissivity was derived from the M-AERI radiance observations during the MOSAiC campaign.

#### 3.2.1. Surface Skin Temperature

**Figure 7:**
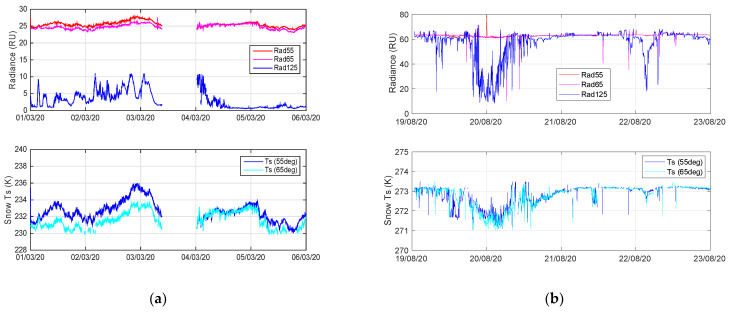
M-AERI surface and sky radiances observations (**upper**) and derived skin temperature (**lower**) for (**a**) 01–05 March 2020 and (**b**) 19–22 August 2020.

**Figure 8:**
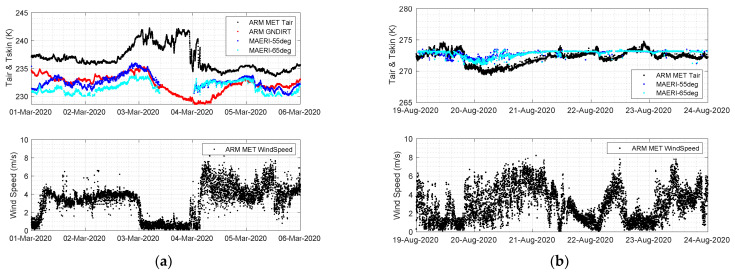
M-AERI T_skin_ compared with ARM GNDIRT T_skin_ and ARM Met T_air_ (**upper**) and ARM Met wind speed (**lower**) for (**a**) 1–5 March 2020 and (**b**) 19–22 August 2020.

The M-AERI summary product variable “longwaveWindowRadiance985_990” contains the mean observed radiance for the narrow atmospheric window region 985–990 cm^−1^ (10.1 μm) for each scan angle. The upper panels of Figure 7 illustrate the M-AERI radiance observations for selected scan angles plotted for the two case study time periods in March and August 2020. The label Rad55 is the radiance from the surface viewing angle at 55° from nadir and the Rad65 is the radiance from the view angle at 65° from nadir. These radiances are represented in Equation (1) by the “up” label. The label Rad125 is the radiance at the view angle 125° from nadir, which is 55° from the zenith and therefore represents the downwelling radiance from the atmosphere which is incident on the sea surface. This is labeled “dwn” in Equation (1). Under the assumptions of Equation (2), the surface skin temperature (at 987.5 cm^−1^) was derived as shown in the bottom panels of Figure 7. Figure 8 compares the M-AERI skin temperature estimates at these two slant angles to ARM MET air temperature and to the ARM downlooking IRT surface temperature for March 2020. Only the MET air temperature was available from August 2020 for comparison. The coincident wind speeds during these time periods are also shown in Figure 7 to assist in the interpretation of results. Additional figures showing radiosonde and ceilometer data for these time periods can be found in Appendix A.

#### 3.2.2. Surface Infrared Emissivity

**Figure 9:**
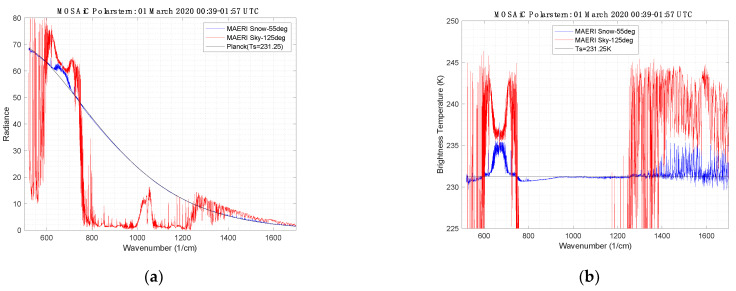
(**a**) M-AERI observation of snow surface emission and sky radiance and (**b**) equivalent blackbody temperature from 1 March 2020 00:39–01:57 UTC.

**Figure 10:**
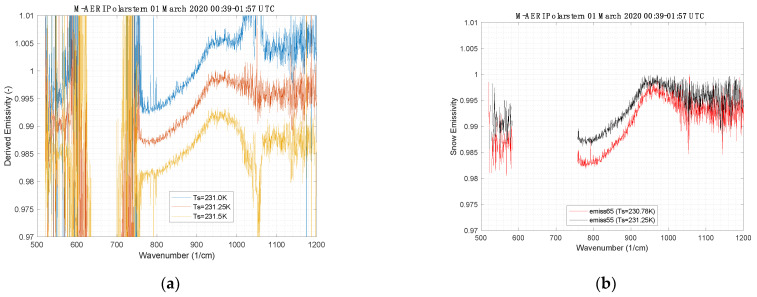
(**a**) M-AERI-derived thermal infrared emissivity at 55° from nadir for three skin temperature guesses and (**b**) the final emissivity for the skin temperature which minimizes atmospheric line structure for the 55° (and 65°) view.

A cloud-free period on 1 March 2020 was used to derive the snow surface infrared emissivity from the M-AERI radiance observations. Figure 9 shows the observed radiance from the 55° downlooking view and the coincident observed radiance from the complimentary 125° view. The same data are plotted in Figure 9 as brightness temperature for later discussion. Using the spectral variance method of [28], the skin temperature that minimizes the atmospheric line structure in the derived infrared emissivity was determined. Figure 10 illustrates the derived emissivity as a function of assumed skin temperature. Note in particular that the residual impact of the ozone region between 1000 and 1100 cm^−1^ is removed from derived emissivity when the correct skin temperature is used. After finding the correct skin temperature, the resulting final spectral infrared emissivity is shown in Figure 10 for the 55° and 65° view angles. Note that the 500–600 cm^−1^ spectral region is considered to be a portion of the far-infrared spectrum, also known as the dirty window.

### 3.3. Satellite Sounder Profile Validation

The NOAA NUCAPS operational profile was validated using observations from the MOSAiC campaign for both vertical temperature and water vapor profiles and retrieved surface parameters.

#### 3.3.1. Temperature and Water Vapor Profile Comparison

**Figure 11:**
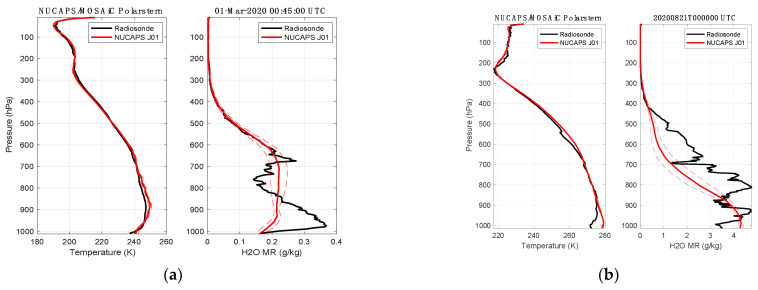
Comparison of NUCAPS versus radiosonde temperature and water vapor for (**a**) 1 March 2020 and (**b**) 21 August 2020. The NUCAPS solid line is the mean of the three closest soundings to the Polarstern and the dashed line is the standard deviation.

NOAA NUCAPS satellite soundings were obtained for each of the dates of the case study time periods and coincident time/space matchups were found with the Polars tern location. The radiosonde closest to the overpass time is compared with the three NUCAPS soundings closest in space to the RV Polarstern. Figure 11 shows an overlay of the radiosondes with the NUCAPS soundings. The NUCAPS mean of the three closest soundings is shown along with the standard deviation among the NUCAPS.

#### 3.3.2. Surface Infrared Temperature and Emissivity Comparison

**Figure 12:**
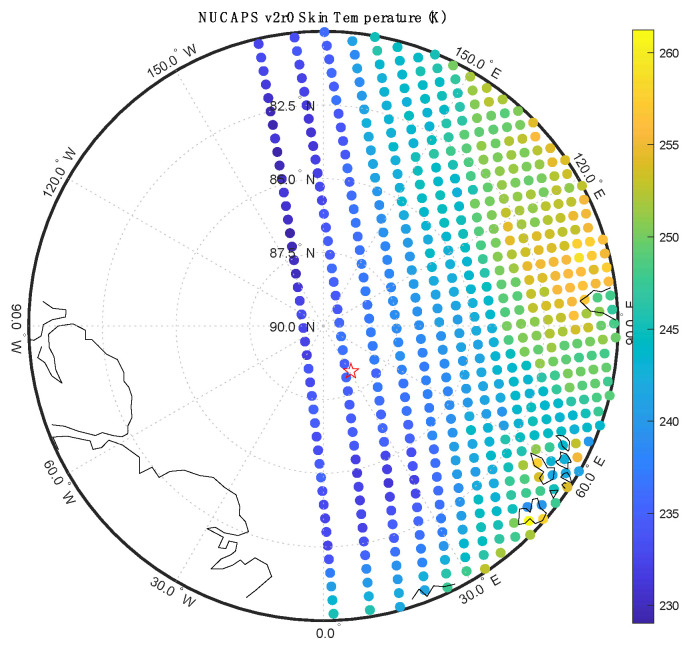
NOAA20 NUCAPS skin temperature map on 1 March 2020 with the RV Polarstern location shown as a red star. The exact time of the RV Polarstern observations was 00:55:49 UTC.

**Figure 13:**
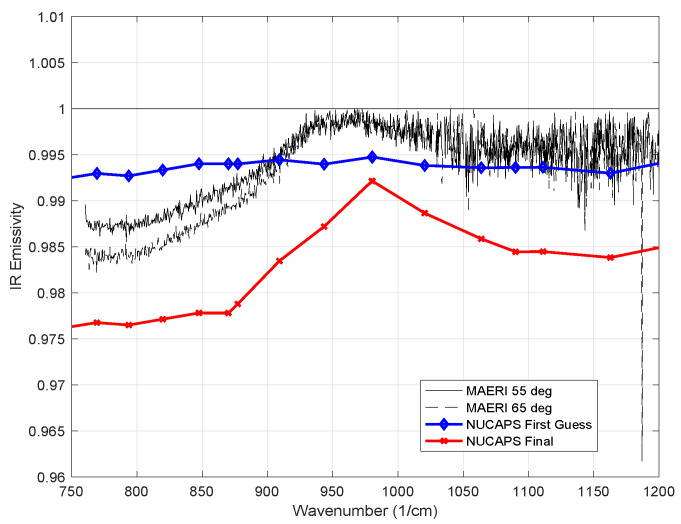
Comparison of NUCAPS v2 mid-infrared emissivity at 45.0° from nadir view angle used in satellite sounding retrieval and the M-AERI measured emissivity at 55° and 65° view angles for 1 March 2020 00:55:49 UTC.

The NOAA NUCAPS sounding files also contain estimates of the surface skin temperature and surface emissivity for the sounding footprint (~50 km diameter). Figure 12 shows the cross-track swath of NUCAPS skin temperature estimates for the first NOAA20 overpass of the RV Polarstern on 1 March 2020. This was the coldest period of the MOSAiC campaign and relatively cloud-free. The NUCAPS skin temperature values were extracted for the closest NUCAPS footprints and compared to validation observations from the ARM GNDIRT and the M-AERI. Table 2 summarizes the coincident comparison. The NUCAPS SkinT value is the 50 km footprint average that contains the RV Polarstern location. The NUCAPS SkinT uncertainty is the spatial standard deviation of the three closest NUCAPS footprint estimates in the same cross-track swath. The three NUCAPS view angles used in the standard deviation are 41.73° (235.20 K), 45.06° (235.69K), and 48.39° (233.04) relative to satellite nadir. The ARM GNDIRT, the M-AERI 55° and M-AERI 65° skin temperature estimates are the instantaneous values closest in time to the satellite overpass at 1 March 2020 00:56 UTC. The uncertainty of GNDIRT and M-AERI shown in Table 2 is the temporal standard deviation over a one hour period centered at the satellite overpass time. A comparison of the NUCAPS infrared emissivity used in the retrieval and the M-AERI measured surface emissivity is shown in Figure 13. Further discussion is reserved for the next section.

## 4. Discussion

The proper interpretation of the results of this paper requires a brief review of the uncertainty of the M-AERI measurements. A recent review of ship-based M-AERI Fourier transform infrared (FTIR) interferometers describes validation over a quarter of a century of remotely sensed sea surface skin temperature measurements [36]. Statistical comparison of M-AERI IR skin temperature measurements against those found in global reanalysis tied to drifting buoys provide one measure of M-AERI accuracy [37]. In this paper, the high absolute accuracy of the surface skin temperature measurements (~0.1 K) is derived using a propagation of errors from the calibration approach of the M-AERI [15]. For calibration, the M-AERI uses a high emissivity blackbody at ambient air temperature with an absolute temperature accuracy of 0.05 K (3-sigma) [16]. When the M-AERI views a target scene that is near ambient temperature, e.g., a horizontal view of the air or a downlooking view of the surface, the radiometric accuracy of the radiance measurement takes full advantage of the high accuracy of the ambient blackbody. The reader is referred to Figure 4 of Knuteson et al. AERI Part II [17] for an illustration of the minimum in radiometric calibration error at ambient temperatures. The sky view radiance measurements have an uncertainty as described in Knuteson et al. [17] of less than 1% of an ambient temperature blackbody radiance for all scene temperatures. The main difference between remote sensing of the ocean surface and snow/ice surfaces is the uncertainty in the knowledge of the infrared emissivity. The M-AERI observations during the MOSAiC campaign are unique due to the simultaneous measurements of surface skin temperature as illustrated in Figure 8 and surface emissivity as illustrated in Figure 10. Propagating uncertainties for the MOSAiC campaign, the M-AERI horizontal air temperature measurements have an absolute temperature uncertainty estimate of 0.1 K (k = 2) and the skin temperature measurements of 0.2 K (k = 2), accounting for uncertainty in the surface emissivity.

**Figure A2:**
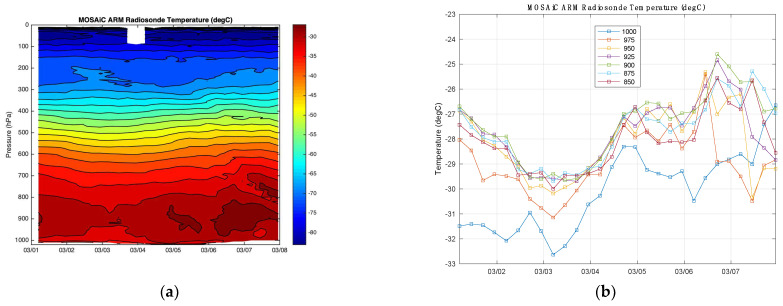
(**a**) Radiosonde time-pressure cross-section and (**b**) boundary layer time series of air temperature at selected boundary layer pressure levels for 1–7 March 2020. Black plus symbols are the M-AERI near-surface air temperatures; blue X symbols are the M-AERI-estimated skin temperatures. High winds and blowing snow prevented normal M-AERI operation after 08:00 UTC on 06 March 2020.

**Figure A6:**
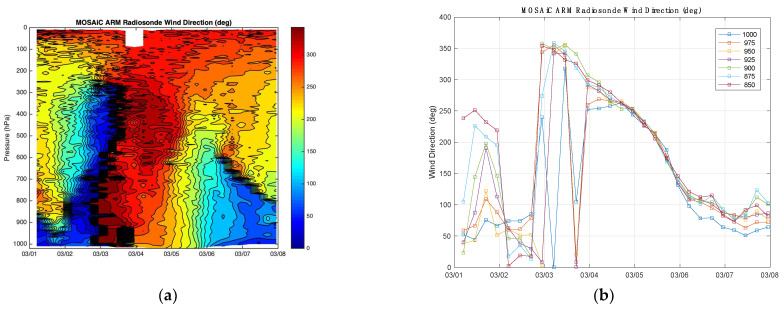
(**a**) Radiosonde time-pressure cross-section and (**b**) boundary layer time series of wind direction at selected boundary layer pressure levels for 1–7 March 2020. Symbols are the same as Figure A2.

**Figure A8:**
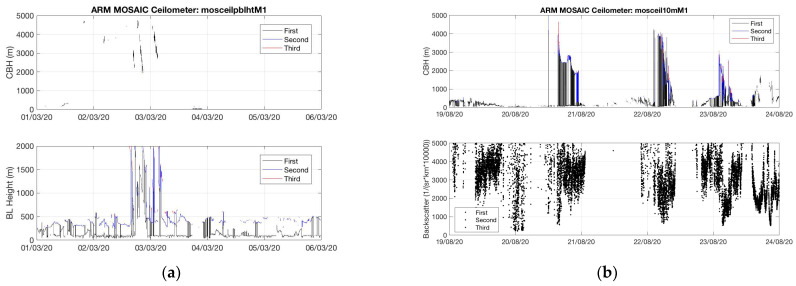
(**a**) ARM Ceilometer CBH and PBL Height for 1–5 March 2020. (**b**) ARM Ceilometer CBH and Backscatter for 19–23 August 2020.

**Figure A9:**
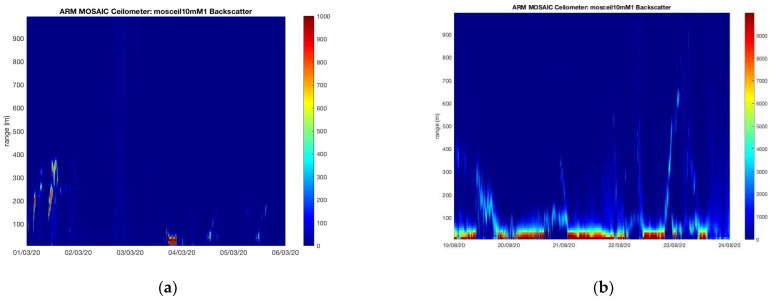
ARM Ceilometer Backscatter time-height cross-section at (**a**) 1–5 March 2020 and (**b**) 19–23 August 2020.

For the March 2020 case, the M-AERI observations of near-surface air temperature shown in Figure 6 are consistent with a temperature inversion with temperatures increasing with height above the snow surface. The DOE ARM MET tower air temperature is colder than the M-AERI horizontal air measurements from the ship by 0.5 K to 1.0 K, which is consistent with the coldest temperature of the air just above the ice pack. Note that the ARM MET tower is some distance (~2 km) from the RV Polarstern (see Figure 2 and Figure 3) and is thus less influenced by the micro-climate near the ship [5]. The time period at the end of 2 March was characterized by a change in wind direction and a slight temperature and moisture increase, see Figure A6, which brought M-AERI observed temperatures at all the observed heights into temporary agreement at the beginning of 3 March. The M-AERI hatch closed during the second half of 3 March, causing a gap in the M-AERI time series until 4 March at 0 UTC, when data resumed. The period of 4–5 March showed the coldest near-surface air temperature and the largest difference between the 95° M-AERI horizontal brightness temperature and the 180° zenith view above the ship. The span of temperatures between the near-surface MET tower and the zenith viewing M-AERI is ~3 K over a range of ~100 m. The radiosonde data time series shown in Figure A2 shows a similar increase in the lapse rate starting 4 March. In contrast, the August 2020 case shown in Figure 6 suggests that all of the M-AERI uplooking views sense the same brightness temperature. This time period was chosen because of the melting seen in Figure 5. The lack of any brightness temperature variation with view scan angle indicates the presence of an isothermal boundary layer from the level of the P-deck on the RV Polarstern to ~100 m above the ship. Meanwhile, the ARM MET tower near-surface air temperature follows the trend of the M-AERI brightness temperature measurements in time but is about 1 K colder when the air temperature is below the freezing point. The air temperatures over this time vary between 270 K and 273 K during the time period. The dip in temperatures from 19 August 20:00 UTC to 20 August 10:00 UTC is consistent with a thinning of the overcast cloud, as evidenced by the ceilometer data shown in Figure A8 and Figure A9, leading to radiative cooling of the surface skin temperature. This is also seen in the M-AERI longwave window air temperature zenith data shown in Figure A1.

The M-AERI surface skin temperature derived using Equation (2) was compared to the ARM GNDIRT in Figure 8 for the March 2020 case study. The differences between the GNDIRT and the M-AERI skin temperature are larger than the estimated uncertainty of the M-AERI measurements. The temperature differences also change with time over the period shown in Figure 8. This time period was relatively clear as evidenced by the ceilometer data shown in Figure A8 and Figure A9. However, the zenith viewing M-AERI brightness temperature at 180° shown in Figure A1 indicates the presence of cirrus cloud for periods of 1–3 March, with a clear period at the beginning of 1 March during the NUCAPS satellite overpass selected for comparison in this paper. The period from 4 March 12 UTC through 5 March has lower downwelling sky radiance. Since Equation (2) accounts for the observed downwelling sky radiance, the presence of clouds on M-AERI-derived skin temperature is negligible. This makes the method chosen for deriving the M-AERI skin temperature robust for most environmental conditions. There does not appear to be a systematic bias between the ARM GNDIRT and the M-AERI T_skin_, but the differences between the ARM GNDIRT- and M-AERI-derived skin temperature are worth further discussion. There does not appear to be any correlation with the wind speed shown in Figure 8; however, comparison to Figure A6 suggests that wind direction may be an important factor in explaining the difference between M-AERI T_skin_ and ARM GNDIRT. A hypothesis that could explain the skin temperature differences is that the RV Polarstern is blocking the free movement of air across the sea surface at the location of the M-AERI observation, thereby changing the radiative cooling at the surface adjacent to the ship [5]. For the August case study, there was no ARM GNDIRT data available, presumably due to difficulty in installing the ARM radiation station during the melting conditions. However, Figure 8 does show the influence of freezing air temperatures on 20 August leading to M-AERI skin temperature values that drop below the freezing point then slowly return to the melting point of water. This suggests that the M-AERI is witnessing a period of the Arctic surface melting, followed by re-freezing, and then subsequent melting again. This time period could be interesting to investigate further in order to more fully characterize the surface radiation effects and the balance between upwelling and downwelling infrared radiation during the melt phase. In this case, a low overcast contributes to melting whereas a higher, thinner cloud promotes re-freezing of the surface.

The derivation of the infrared surface emissivity spectrum from the M-AERI observations provided a unique opportunity to obtain measurements that constrain models of snow and ice radiation in a realistic Arctic environment. The interpretation of R^dwn^ in Equation (2) as a hemispheric flux follows from the assumption that the snow surface is diffuse. The 125° sky view is used as an approximation of the hemispheric downwelling flux from a uniform sky. If the surface has a specular component, then the appropriate downwelling angle is also 125° since the angle of incidence at the surface equals the angle of reflection. Therefore, the 55° view measurement of emissivity is robust with respect to the surface being diffuse or specular or some weighted average. The emissivity shown in Figure 10 is effectively a point measurement at two specific view angles; therefore, extrapolation to larger scenes and other view angles could be problematic.

The NUCAPS profile assessment shown in Figure 11 is straightforward and the results are consistent with previous work [24,25]. The temperature profile agreement for the 1 March 2020 case is excellent with only a small error in the inversion strength at 900 hPa but very good agreement near the sea surface. The second case study contains low overcast; thus, the deviation of the satellite sounding below the cloud base at 900 hPa is not expected to agree. For altitudes above 900 hPa, the agreement between the NUCAPS temperature retrieval and the radiosonde measured air temperature is excellent. In contrast, the water vapor comparison illustrates the lower vertical resolution of the NUCAPS sounding, which smooths through much of the fine vertical structure found in the radiosonde dew point measurements. However, there are low vertical resolution water vapor biases in Figure 11 for 21 August that suggest a more careful assessment should be carried out for this case. A comprehensive study of NUCAPS temperature and water vapor profiles over the entire MOSAiC campaign data is recommended.

The NUCAPS skin temperature comparison shown in Table 2 is remarkably good considering that the NUCAPS represents a 50 km diameter circle containing the RV Polarstern, whereas the M-AERI observation is less than 50 cm in diameter. It is worth noting that the view angle for the NUCAPS of 45° is similar to the view angle for the M-AERI at 55° for this case. The uncertainty estimates shown in Table 2 are an attempt to capture the spatial and temporal uncertainty but do not include estimates of the absolute radiometric accuracy. The M-AERI absolute accuracy is estimated to be about 0.2 K (k = 2); therefore, the +4.5 K higher value of NUCAPS skin temperature requires further discussion. The derived skin temperature of NUCAPS is influenced by the emissivity spectrum assumed in the retrieval code. Figure 13 overlays the emissivity used in NUCAPS version 2.0 for a first guess and in the final retrieval for the coincident observation with the emissivity derived from M-AERI for the same date and time. The first guess is nearly constant with wavelength but is similar in magnitude to the average of the M-AERI-derived infrared emissivity spectrum. The NUCAPS final emissivity shows some of the curvature with wavenumber that the M-AERI emissivity spectrum also exhibits but the magnitude is lower. This lower NUCAPS final emissivity could explain why the NUCAPS surface temperature is larger than the M-AERI surface temperature. In infrared radiative transfer theory, an error in surface skin temperature is inversely correlated with an error in surface emissivity. This suggests that a better constrained emissivity retrieval within NUCAPS could improve the surface temperature estimate for the 1 March case. The same argument applies to the interpretation of the GNDIRT temperature; the emissivity assumed in the GNDIRT measurement is not known to the authors but it is likely that a lower emissivity was assumed than that measured by the M-AERI for the 1 March snow conditions. A higher emissivity of the ARM GNDIRT might bring the skin temperature into better overall agreement with M-AERI also.

## 5. Conclusions

The 2019–2020 MOSAiC campaign brought state-of-the-art measurements to the Arctic sea ice to observe the ocean/sea ice/atmosphere for a complete calendar year. The U.S. DOE contributed an ARM Mobile Facility suite of sensors to MOSAiC. One of the unique contributions from ARM was a Marine-AERI sensor with slant angle views of the sky, horizon, and sea ice surface. The M-AERI infrared spectral radiance measured air temperatures within the lowest 1000 m of the atmospheric planetary boundary layer under a variety of meteorological conditions. The M-AERI skin temperature provides a useful complement to other surface radiation sensors deployed as part of the MOSAiC campaign. The M-AERI measurement of surface infrared emissivity is also important for validation of remote sensing from infrared sensors of satellite and airborne platforms. Preliminary comparison with the NOAA NUCAPS operational satellite sounding shows good agreement while suggesting areas for future research.

## Figures and Tables

**Table 1:** M-AERI viewing conditions.

Date Range	Atmospheric Conditions	Surface Conditions
**13-January-2020** **10-March-2020**	Mostly windy conditions with cloudy skies	First day to March 10th is 100% snow
**11-March-2020** **19-April-2020**	Snowy and windy conditions through this time period	Starting March 11th, there is a mixture of ice-water-snow
**18-July-2020** **31-July-2020**	Foggy to clear conditions	Data gap after April, ice transition into open water
**21-August-2020** **20-Septemter-2020**	Snowy, low visibility	Ice and snow, followed by melt ponds

**Table 2:** Validation of surface skin temperature from NUCAPS on 1 March 2020 00:55:49 UTC with 50 km diameter sounding footprint at Latitude: 88.0895, Longitude: 23.9789, and CrIS View Angle: 45.06.

NUCAPS SkinT (K)	ARM IRT (K)	M-AERI 55°	M-AERI 65°
235.692 ± 1.151	234.300 ± 0.084	231.3244 ± 0.066	230.839 ± 0.109

**Figure A3:**
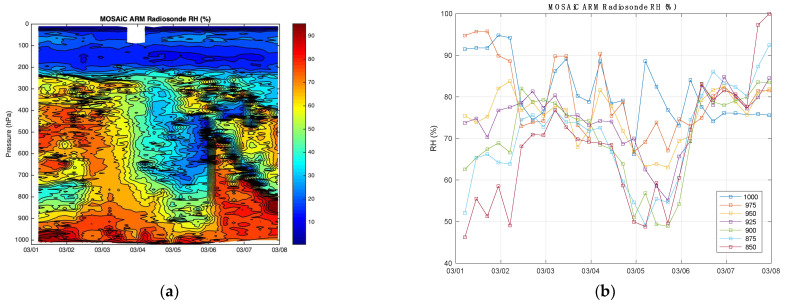
(**a**) Radiosonde time-pressure cross-section and (**b**) boundary layer time series of air relative humidity at selected boundary layer pressure levels for 1–7 March 2020. Symbols are the same as A2.

**Figure A4:**
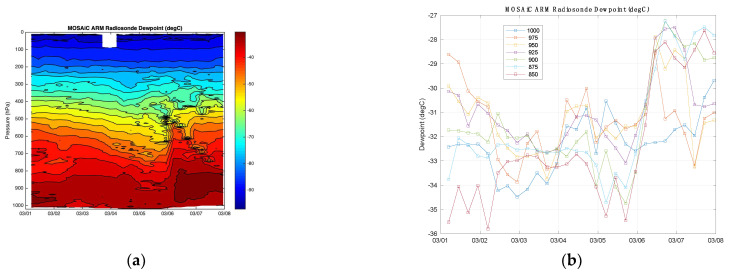
(**a**) Radiosonde time-pressure cross-section and (**b**) boundary layer time series of water vapor dewpoint temperature at selected boundary layer pressure levels for 1–7 March 2020. Symbols are the same as A2.

**Figure A5:**
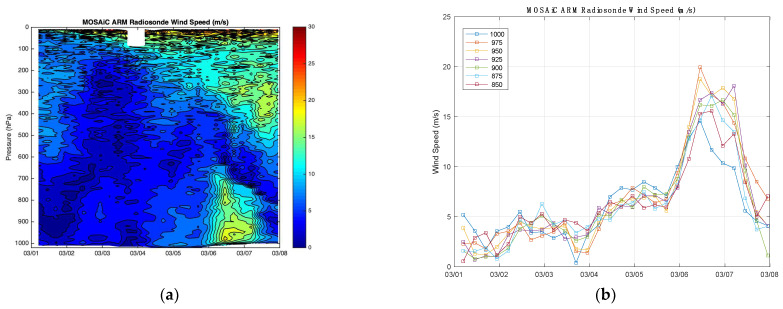
(**a**) Radiosonde time-pressure cross-section and (**b**) boundary layer time series of wind speed at selected boundary layer pressure levels for 1–7 March 2020. Symbols are the same as A2.

**Figure A7:**
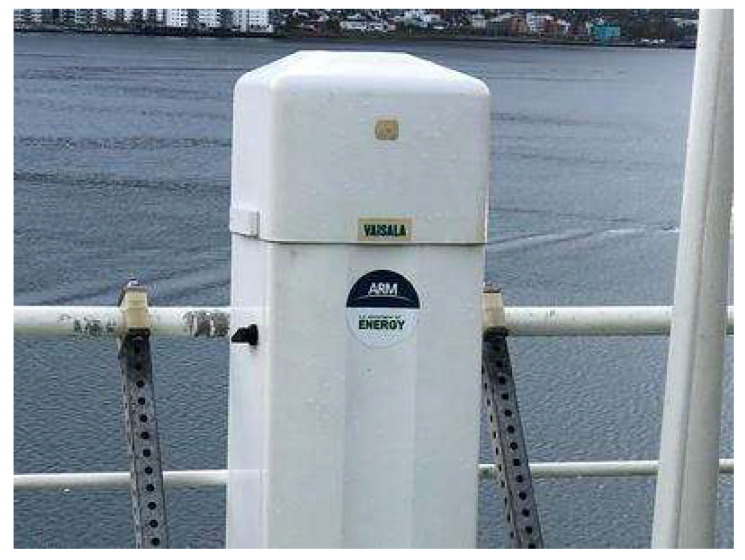
DOE ARM Ceilometer. [Image courtesy of the U.S. Department of Energy Atmospheric Radiation Measurement (ARM) user facility].

## Data Availability

The data presented in this study are openly available in U.S. Department of Energy. Atmospheric Radiation Measurement (ARM) user facility archive. 2019. Atmospheric Emitted Radiance Interferometer (AERISUMMARY). 1 October 2019 to 30 September 2020, ARM Mobile Facility (MOS) MOSAiC (Drifting Obs—Study of Arctic Climate); AMF2 (M1). Compiled by J. Gero, H. Revercomb, D. Turner, J. Taylor, R. Garcia, D. Hackel, B. Ermold and K. Gaustad. ARM Data Center. Data set accessed 1 June 2022 at http://dx.doi.org/10.5439/1025146.

## Appendix A

This appendix contains meteorological information that is useful in the interpretation of the observed M-AERI radiances. The measured brightness temperatures from the M-AERI at selected wavenumbers are shown in Figure A1 for the two cases of interest in this paper. Note that the surface layer and elevated layer brightness temperature are mainly sensitive to the air temperature for each M-AERI scan angle. In contrast, the longwave window brightness temperature directly views the surface emission (and reflection) in the downlooking angles (55 and 65°) and the cloud base (or clear sky radiance) in the uplooking views (115, 125, and 180). The 95° view is nearly horizontal to the RV Polarstern and represents a long path mean air temperature. The radiosonde temperature and water vapor and wind time-height cross-sections are shown in Figure A2, Figure A3, Figure A4, Figure A5 and Figure A6. Additionally, time series of the radiosonde data interpolated to selected pressure levels are shown. Note that the 1000 hPa level is always above the surface for this time period. To assist in the interpretation of the M-AERI radiances, the cloud conditions above the RV Polarstern were characterized using the ARM ceilometer shown in Figure A7. The ceilometer cloud base height and PBL height as shown in Figure A8 for the two time periods. The ARM ceilometer backscatter cross-section shown in Figure A9. The ceilometer data indicate that the March case was mostly cloud-free with some mid-level clouds, while the August case had mostly low overcast cloud conditions with some cloud breaks.
